# Highly Sensitive Diode-Based Micro-Pirani Vacuum Sensor with Low Power Consumption

**DOI:** 10.3390/s19010188

**Published:** 2019-01-07

**Authors:** Debo Wei, Jianyu Fu, Ruiwen Liu, Ying Hou, Chao Liu, Weibing Wang, Dapeng Chen

**Affiliations:** 1 Smart Sensing Research and Development Centre, Institute of Microelectronics, Chinese Academy of Sciences, Beijing 100029, China; weidebo@ime.ac.cn (D.W.); houying@ime.ac.cn (Y.H.); liuchao@ime.ac.cn (C.L.); wangweibing@ime.ac.cn (W.W.); dpchen@ime.ac.cn (D.C.); 2 School of Electronic, Electrical and Communication Engineering, University of Chinese Academy of Sciences, Beijing 100029, China

**Keywords:** highly sensitive, low power consumption, diode, micro-Pirani vacuum sensor

## Abstract

Micro-Pirani vacuum sensors usually operate at hundreds of microwatts, which limits their application in battery-powered sensor systems. This paper reports a diode-based, low power consumption micro-Pirani vacuum sensor that has high sensitivity. Optimizations to the micro-Pirani vacuum sensor were made regarding two aspects. On the one hand, a greater temperature coefficient was obtained without increasing power consumption by taking advantage of series diodes; on the other hand, the sensor structure and geometries were redesigned to enlarge temperature variation. After that, the sensor was fabricated and tested. Test results indicated that the dynamic vacuum pressure range of the sensor was from 10^−1^ to 10^4^ Pa when the forward bias current was as low as 10 μA with a power consumption of 50 μW. Average sensitivity was up to 90 μV/Pa and the sensitivity of unit power consumption increased to 1.8 V/W/Pa. In addition, the sensor could also work at a greater forward bias current for better sensor performance.

## 1. Introduction

Micro-Pirani vacuum sensors are of vital importance in vacuum pressure measurement in modern society. In recent decades, scholars have committed to the development of high-performance micro-Pirani vacuum sensors that have a large dynamic vacuum pressure range, small size, and are complementary metal oxide semiconductor (CMOS) compatible. With the development of micromachining technology, various microelectromechanical systems (MEMS)-based micro-Pirani vacuum sensors with a complex structure and small size have been designed and fabricated [1,2,3]. These micro-Pirani vacuum sensors are particularly suitable to extremely miniaturized devices, and they offer the possibility of performing in situ vacuum pressure monitoring inside sealed MEMS packages of inertial sensors, such as gyroscopes, accelerometers, and inertial measurement units [4,5,6,7,8,9].

Great research efforts have been devoted to enlarging the dynamic vacuum pressure range. A micro-Pirani vacuum sensor with five-decade operating vacuum pressure range has been developed with the help of mutual heat transfer, and its detection limit has been expanded to 3 × 10^−1^ Pa [10]. The micro-Pirani vacuum sensor with triple heat sinks has contributed to a larger dynamic vacuum pressure range, from 0.02 to 500 Torr (from 2.66 to 6.65 × 10^4^ Pa) [11]. Laser-induced heating is applied to expand the dynamic vacuum pressure range thus that the lower limit of vacuum pressure measurement is as low as 10^−6^ Torr (1.33 × 10^−4^ Pa) [12]. For beyond atmospheric pressure, micro-Pirani vacuum sensors have also been exploited, and some of these show good sensitivity to vacuum pressure up to 7 × 10^5^ Pa [13,14]. Apart from a larger dynamic vacuum pressure range, smaller micro-Pirani vacuum sensor size is also an important parameter. A nano-Pirani sensor has been fabricated by a focused ion beam and its size is 30 × 3 μm [15]. In addition, a 5-μm-long single-walled carbon nanotube has been used in micro-Pirani vacuum sensors, which reduced the sensor size to 63 × 2 μm [16].

In addition to large dynamic vacuum pressure range and small sensor size, much attention has been focused on sensitivity and power consumption due to the application of micro-Pirani vacuum sensors in portable measuring equipment. Increasing the area of gaseous heat loss is an effective way of improving sensitivity. A hollow heater design has been developed that obtained a sensitivity of unit power consumption of 8.15 × 10^3^ K/W/Torr (0.00187 V/W/Pa at current of 10 mA) in the pressure range of 0.2–200 Torr (26.6 to 2.66 × 10^4^ Pa) with a power consumption of 127.59 μW [17]. A high aspect ratio design has helped improve the sensitivity of unit power consumption to 117 K/W/Pa (0.012 V/W/Pa) in the vacuum pressure range of 1–10^3^ Pa with power consumption of less than 40 mW [18]. Another important method is micro-hotplate (MHP) technology, for which the sensitivity of a micro-Pirani vacuum sensor has reached 230 μV/Pa (0.02 V/W/Pa) in the vacuum pressure range of 1–10^2^ Pa with power consumption of 4900 μW [19]. Previous works have made great contributions to the development of micro-Pirani vacuum sensors, especially regarding theoretical analysis and structural design [20,21,22]. However, for complex sensor systems, the power consumption of a single micro-Pirani vacuum sensor is still too high and needs to be reduced, especially for battery-powered sensor systems.

In this paper, a highly sensitive, diode-based micro-Pirani vacuum sensor with low power consumption is presented. The sensor works at a constant forward bias current, and the voltage drop across the diode varies with vacuum pressure because of the temperature coefficient of the diode. To improve sensitivity and reduce power consumption, a greater temperature coefficient has been obtained by taking advantage of series diodes, and larger temperature variation of the micro-Pirani vacuum sensor has been achieved by making optimizations to the sensor structure. The remainder of the paper is presented as follows: First, the basic principle of the diode-based micro-Pirani vacuum sensor is introduced; next, the simulation and optimizations are presented; finally, the outcome of the fabrication and testing of the highly sensitive micro-Pirani vacuum sensor is described. Test results indicate that the presented series-diode-based micro-Pirani vacuum sensor displays high sensitivity and low power consumption and is a good candidate for vacuum pressure monitoring.

## 2. Basic Principle of the Diode-Based Micro-Pirani Vacuum Sensor

**Figure 1:**
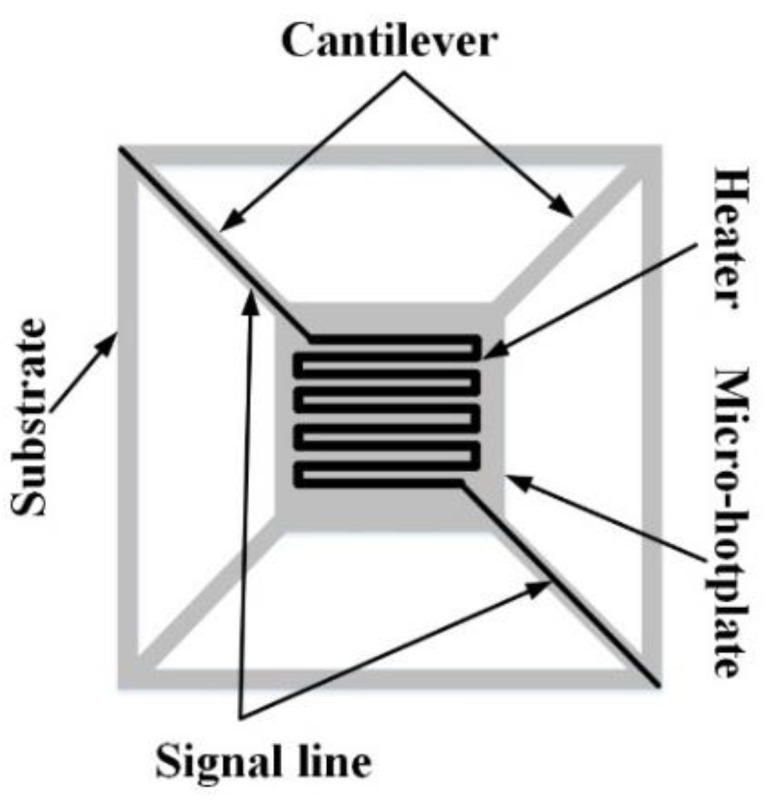
Classical design of a micro-hotplate (MHP) in a micro-Pirani vacuum sensor.

Micro-Pirani vacuum sensors are based on the fact that the heat loss of an MHP to its ambient environment through gas conduction is proportional to the vacuum pressure in a vacuum system [23]. Figure 1 shows the classical design of a vacuum sensor based on an MHP for improving vacuum sensor performance [24]. The MHP is supported by four cantilevers and these cantilevers are connected to a substrate. The heater, which is usually a thermistor, is located in the MHP, and signal lines are embedded in the cantilevers.

The temperature of the MHP rises due to the electric heating power of the heater, however, the heat loss through the cantilevers, gas, and radiation decreases the MHP’s temperature. A balanced state is reached in the end, and the relationship can be expressed as [25]

(1) $$Q_{power}=Q_{gas}(P)+Q_{solid}+Q_{rad}$$

where *Q_power_* is the electric heating power of the heater, *Q_gas_* is the gaseous heat loss, P is the vacuum pressure, *Q_solid_* is the solid heat loss, and *Q_rad_* is the radiation heat loss. Among the three ways of heat loss, gaseous heat loss depends on vacuum pressure, thus the equilibrium temperature *T_e_* of the MHP is related to the vacuum pressure. Therefore, the temperature variation of the MHP, that is, the temperature difference between the equilibrium temperature and the environment temperature *T_a_*, depends on the vacuum pressure:

(2) $$\Delta T(P)=T_{e}(P)-T_{a}.$$

**Figure 2:**
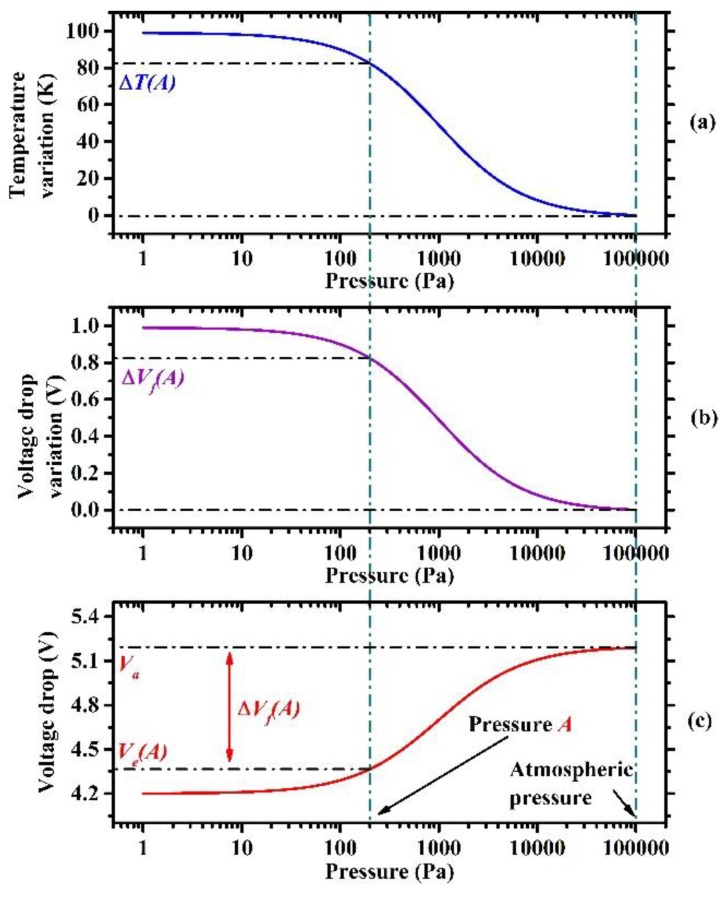
(**a**) Temperature variation of a micro-hotplate (MHP) rises with the decrease in vacuum pressure because of the reduced gaseous heat loss, (**b**) voltage drop variation of six series diodes rises with temperature variation, (**c**) voltage drop across six series diodes falls with decreasing vacuum pressure as a result of the negative temperature coefficient of diode.

In micro-Pirani vacuum sensors, this MHP temperature variation is used to measure vacuum pressure [26]. For the diode-based micro-Pirani vacuum sensor, the diode is located in the MHP and works as both a heater and detector at a constant forward bias current. The voltage drop across the diode varies with the MHP temperature because of the diode temperature coefficient. The voltage drop variation is expressed as

(3) $$\Delta V_{f}(P)=\left|V_{e}(P)-V_{a}\right|=\Delta T(P)\left|\frac{dV_{f}}{dT}\right|$$

where ∆*V_f_* is the voltage drop variation, *V_e_* is the voltage drop across the diode with the equilibrium temperature, *V_a_* is the voltage drop across the diode with the environment temperature, and **|***dV_f_*/*dT*| is the temperature coefficient of the diode. When the vacuum pressure changes from atmospheric pressure to pressure *A*, as shown in Figure 2, the temperature variation of the MHP is Δ*T*(*A*) in Figure 2a, the voltage drop variation generated by the temperature variation is Δ*V_f_*(*A*) in Figure 2b, and the measured voltage drop varies from *V_a_* to *V_e_*(*A*) in Figure 2c. The voltage drop falls with decreasing vacuum pressure because of the negative temperature coefficient of the diode and the rising temperature variation in the MHP. Voltage drop variation indicates the response of the micro-Pirani vacuum sensor to a certain vacuum pressure in the dynamic vacuum pressure range. It is usually expected to be as large as possible, and the sensitivity is therefore improved. There are two ways to simultaneously reduce power consumption and enlarge voltage drop variation: Larger temperature variation and a greater temperature coefficient.

## 3. Simulation and Optimization of Micro-Pirani Vacuum Sensor

### 3.1. Greater Temperature Coefficient without Increasing Power Consumption

**Figure 3:**
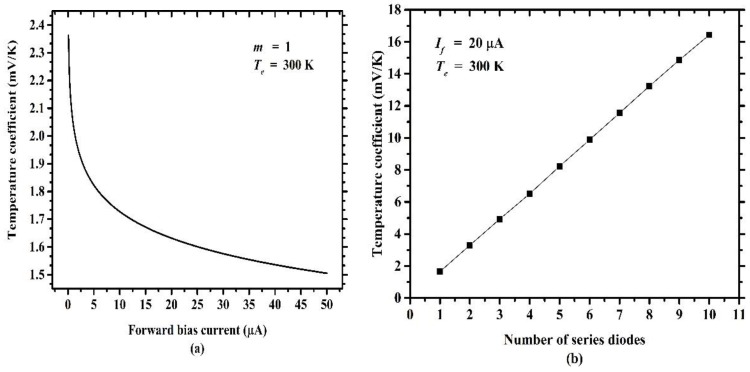
Temperature coefficient dependence on (**a**) forward bias current and (**b**) number of series diodes.

Temperature-sensitive materials are usually used in traditional micro-Pirani vacuum sensors, but the diode-based micro-Pirani vacuum sensor replaces it with a diode by taking advantage of the temperature coefficient of the diode. The relationship between the forward bias current *I_f_* and the applied forward bias voltage *V_f_* of series diodes is given in the following approximate equations [27,28]:

(4) $$I_{f}=I_{s}(T_{e})\left\{\exp\left(\frac{q\frac{V_{f}}{m}}{nkT_{e}}\right)-1\right\}\approx I_{s}(T_{e})\exp\left(\frac{q\frac{V_{f}}{m}}{nkT_{e}}\right)$$

(5) $$I_{s}(T_{e})=qA\left(\frac{D_{n}n_{p0}}{L_{n}}+\frac{D_{p}p_{n0}}{L_{p}}\right)\propto AT_{e}{}^{3+\frac{\gamma }{2}}\exp\left(-\frac{E_{g}}{kT_{e}}\right)$$

where *I_s_* is the saturation current of the diode, *n* is the ideal factor of about 1, *m* is the number of series diodes, and *k* is Boltzmann constant. *q* and *A* are the elementary charge and area of the diode, *n_p_*_0_ and *p_n_*_0_ are minority carriers in *n*-type and *p*-type regions, *D_n_* and *D_p_* are diffusion coefficients of electron and hole, and *L_n_* and *L_p_* are diffusion lengths of electron and hole, respectively. Further, *γ* is a constant and *E_g_* is the energy gap of Si. Combining Equations (4) and (5), the temperature coefficient can be derived as

(6) $$\left|\frac{dV_{f}}{dT}\right|=\alpha \frac{mk}{q}\left\{\left[\left(3+\frac{\gamma }{2}\right)+\frac{E_{g}}{kT_{e}}\right]-\ln\left[\frac{I_{f}\exp\left(E_{g}/kT_{e}\right)}{AT_{e}{}^{3+\gamma /2}}+1\right]\right\}$$

where *α* is a coefficient. Equation (6) indicates that the temperature coefficient is proportional to the number of series diodes. More series diodes can enlarge the temperature coefficient thus that greater voltage drop variation can be achieved, which is similar to prolonging the length of temperature-sensitive material. In addition, the temperature coefficient of the diode is a function of the forward bias current and can be increased by reducing the forward bias current. Figure 3 shows the simulated results of temperature coefficient dependence on the forward bias current and the number of series diodes. It can be seen from Figure 3a that the temperature coefficient rises as the forward bias current decreases. From the point of view of enlarging the temperature coefficient, this can be accomplished by reducing the forward bias current, which is also helpful for reducing power consumption. Figure 3b shows another way of enlarging the temperature coefficient by increasing the number of series diodes.

**Figure 4:**
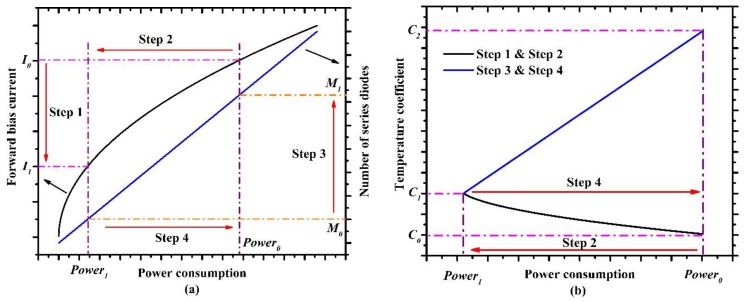
(**a**) Detailed process of obtaining a greater temperature coefficient with the same power consumption. (**b**) Temperature coefficient is improved from *C*_0_ to *C*_1_ by reducing the forward bias current and is further improved from *C*_1_ to *C*_2_ by increasing the number of series diodes.

These two methods—lowering the forward bias current and increasing the number of series diodes—of enlarging the temperature coefficient can be combined for a greater temperature coefficient without increasing power consumption. Figure 4a shows the detailed process of how to achieve a greater temperature coefficient with the same power consumption. Step 1: The forward bias current is cut down from *I*_0_ to *I*_1_. As a result, which is shown as Step 2, the power consumption is reduced from *Power*_0_ to *Power*_1_ because of the reduced forward bias current. However, a strengthened temperature coefficient is achieved. Step 3: The number of series diodes is increased from *M*_0_ to *M*_1_, which contributes to the further improvement of the temperature coefficient. Step 4: More series diodes increase the power consumption back to where it was, which varies from *Power*_1_ to *Power*_0_. Figure 4b shows the results of how the temperature coefficient varied during the detailed process. The reduced forward bias current led to the increase of the temperature coefficient from *C*_0_ to *C*_1_, and then the temperature coefficient changed from *C*_1_ to *C*_2_ because of the increasing number of series diodes. Overall, a greater temperature coefficient was obtained with the same power consumption. Considering the operating voltage, we decided to use six series diodes with a low forward bias current for this work.

### 3.2. Larger Temperature Variation by Sensor Structure Optimization

**Figure 5:**
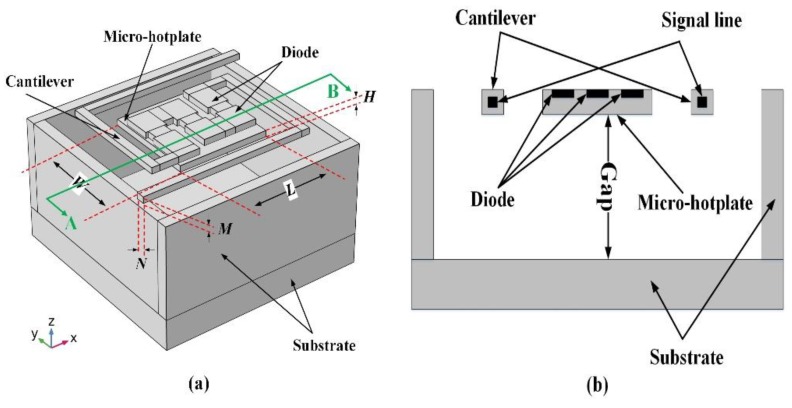
(**a**) Schematic and (**b**) cross-sectional views of the redesigned micro-Pirani vacuum sensor structure.

In addition to the temperature coefficient of six series diodes, temperature variation is another factor that influences the voltage drop variation according to Equation (3). Under the condition of the same power consumption and vacuum pressure, temperature variation depends on the structure of the micro-Pirani vacuum sensor. Compared with traditional MHP-based micro-Pirani vacuum sensors, sensor structure optimizations were made for larger temperature variation. One aspect was to minimize the number of cantilevers from the traditional four to two, which mainly reduced the solid heat loss. Another aspect was to redesign the sensor geometries to further improve the temperature dependence on vacuum pressure. Figure 5a shows the schematic view of the redesigned micro-Pirani vacuum sensor structure, and Figure 5b shows the cross-sectional view along the A–B direction shown in Figure 5a. Two cantilevers connected the substrate and MHP, and the MHP was suspended over the substrate. Series diodes were embedded in the MHP, and its signal was connected out by signal lines made on cantilevers.

**Figure 6:**
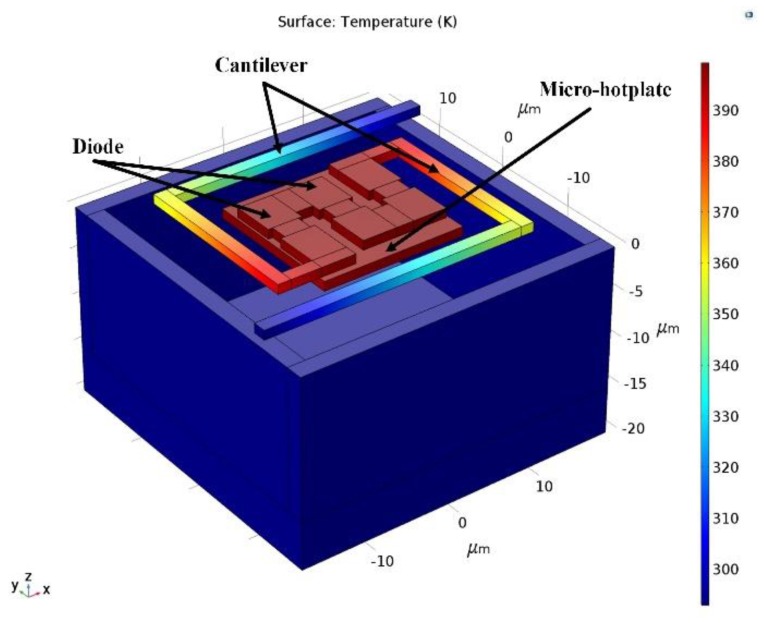
Temperature variation distribution of the micro-Pirani vacuum sensor.

Figure 6 shows the simulated result of temperature distribution in the structure of the micro-Pirani vacuum sensor in a certain vacuum pressure. It can be seen that the MHP had the highest temperature, and the temperature went down along the cantilevers to the temperature of the substrate, which was the environment temperature. The temperature variation of MHP led to voltage drop variation across the series diodes.

Based on this sensor structure, sensor geometries were redesigned for further larger temperature variation, and the heat generation and heat loss of the micro-Pirani vacuum sensor were analyzed. Series diodes were operated at a constant forward bias current, and electric heating power was determined by the self-heating effect of the series diodes. In a balanced state, electric heating power equals heat loss, which is expressed as Equation (1). According to Fourier’s law of heat conduction and the Stefan–Boltzmann law, the expression for this micro-Pirani vacuum sensor is [26]

(7) $$\lambda_{g}(P)WL\frac{T_{e}(P)-T_{a}}{G}+2\lambda_{s}MN\frac{T_{e}(P)-T_{a}}{L_{can}}+2\varepsilon \delta WL\left[T_{e}{\left(P\right)}^{4}-T_{a}^{4}\right]=I_{f}^{2}\left[\frac{V_{f}}{I_{f}}-\left|\frac{dV_{f}}{I_{f}dT}\right|\left(T_{e}(P)-T_{a}\right)\right]$$

where *λ_g_* is the gas thermal conductivity that is dependent on vacuum pressure, *W* is the width of the MHP, *L* is the length of the MHP, *G* is the gap between the MHP and the substrate, *λ_s_* is the thermal conductivity of the cantilever, *M* is the thickness of the cantilever, *N* is the width of the cantilever, *L_can_* is the length of the cantilever from the MHP to the substrate, *ε* is the effective blackbody emissivity of the MHP, and *δ* is the Stefan–Boltzmann constant. Then, temperature variation under a vacuum pressure was approximated as

(8) $$\Delta T(P)=T_{e}(P)-T_{a}\approx \frac{I_{f}V_{f}}{I_{f}\left|\frac{dV_{f}}{dT}\right|+2\varepsilon \delta WL+\frac{2\lambda_{s}MN}{L_{can}}+\frac{\lambda_{g}(P)WL}{G}}.$$

**Figure 7:**
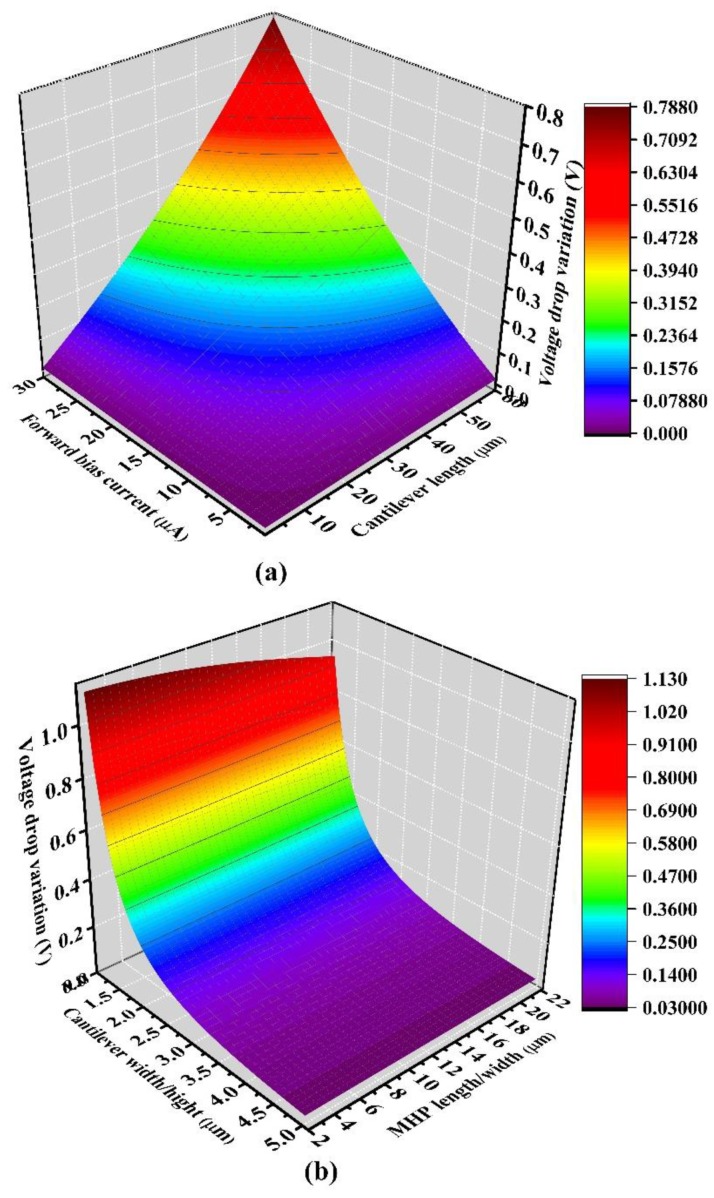
Temperature variation (**a**) is proportional to forward bias current, and cantilever length (**b**) is inversely proportional to length/width of the MHP and the width/thickness of the cantilever.

It can be seen from Equation (8) that temperature variation is proportional to forward bias current. Here, the temperature variation was inversely proportional to the cross-sectional area of the cantilever and the area of the MHP, which were *MN* and *WL*, respectively. Figure 7a,b shows the simulated temperature variation of the six series diodes dependence on the forward bias current, cantilever length, MHP width/length, and cantilever width/thickness. These relationships shown in Figure 7 and Equation (8) can be explained by the mechanism of heat generation and heat loss. Greater electric heating power is obtained and thus more heat is generated when a greater forward bias current is applied. Having a longer cantilever, less cross-sectional area of the cantilever, and a smaller MHP area reduces heat loss, and all of these factors lead to more heat accumulated in the MHP, which behaves according to the larger temperature variation. To obtain a larger temperature variation, the area of the MHP and the cross-sectional area of the cantilever should be reduced as much as possible.

However, a large MHP area would increase gaseous heat loss between the MHP and the substrate; so, to improve the temperature variation dependence on vacuum pressure, it should be kept as large as possible. In order to ensure structural strength, the cross-sectional area of the cantilevers cannot be reduced indefinitely. The redesigned geometries after optimization of the micro-Pirani vacuum sensor are listed in Table 1.

## 4. Fabrication and Experiments of the Micro-Pirani Vacuum Sensor

**Figure 8:**
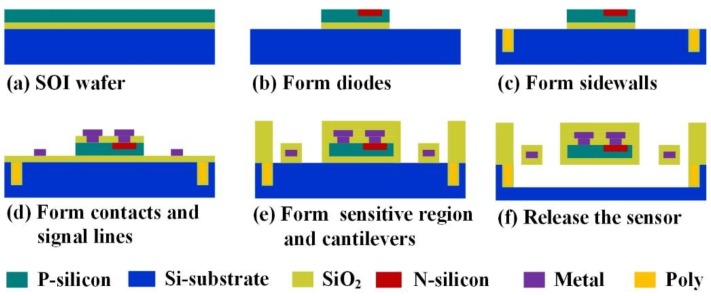
Fabrication process of the micro-Pirani vacuum sensor.

After design, simulation, and optimization, the micro-Pirani vacuum sensor with six series diodes was fabricated with a CMOS-compatible process, which is shown in Figure 8. The detailed process is as follows: (a) The structure was fabricated with a silicon-on-insulator (SOI) wafer, and the top Si layer of the wafer was *p*-type <100>, with the square resistance of 8~12 Ω. (b) The top Si layer was the first ion implanted with boron fluoride (BF_3_) on a doping concentration of 10^13^ at the power of 100 keV, then the second ion was implanted with phosphorus on a doping concentration of 3 × 10^14^ at the power of 100 keV and etched to form the diodes. (c) The Si substrate was patterned by deep reactive ion etching (DRIE) and filled with silicon oxide (SiO_2_) and poly Si to protect the sidewalls of the cavity from being etched. (d) Al was sputtered and patterned thus that the diodes were in series and their signals could be outputted. (e) The sensitive region and cantilevers were formed by deposition and etching of plasma-enhanced chemical vapor deposition (PECVD) SiO_2_. (f) The structure was released by front-side xenon difluoride (XeF_2_) dry etching.

**Figure 9:**
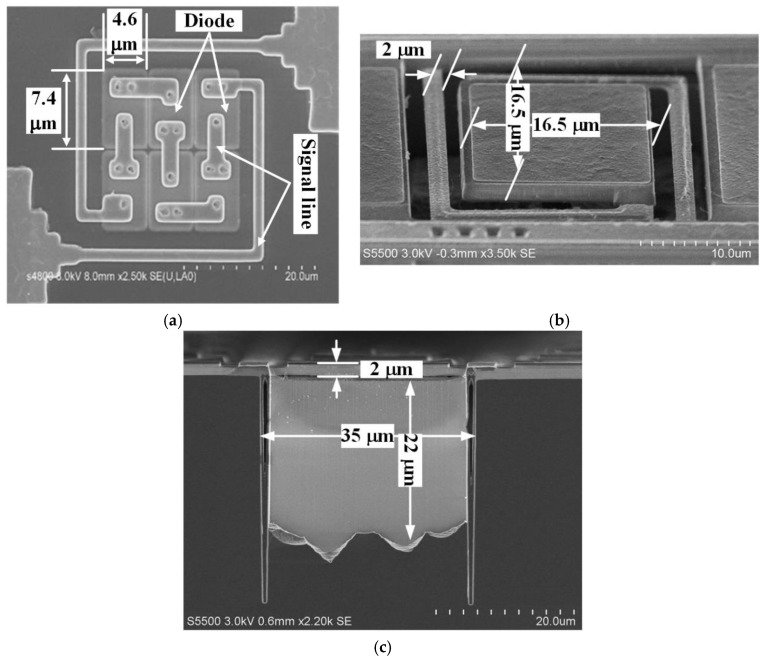
(**a**) SEM image of six series diodes. (**b**) Top and (**c**) cross-sectional views of the fabricated micro-Pirani vacuum sensor.

Figure 9a shows the scanning electron microscope (SEM) image of the series diodes before they were covered by SiO_2_, and it can be seen that the size of a single diode was 7.4 × 4.6 μm. Figure 9b,c shows the top- and cross-sectional-view SEM images of the fabricated micro-Pirani vacuum sensor, respectively. The size of the sensor excluding the area of the pad was 35 × 35 μm, and the size of the sensitive region was 16.5 × 16.5 μm. In addition, the released gap between the MHP and the substrate was about 22 μm. The XeF_2_ etched the Si substrate underneath the sensitive region through the clearance on both sides of the cantilever, and this led to the rough surface of the substrate underneath the MHP. However, this can be improved or even eliminated by optimizing the fabrication process.

**Figure 10:**
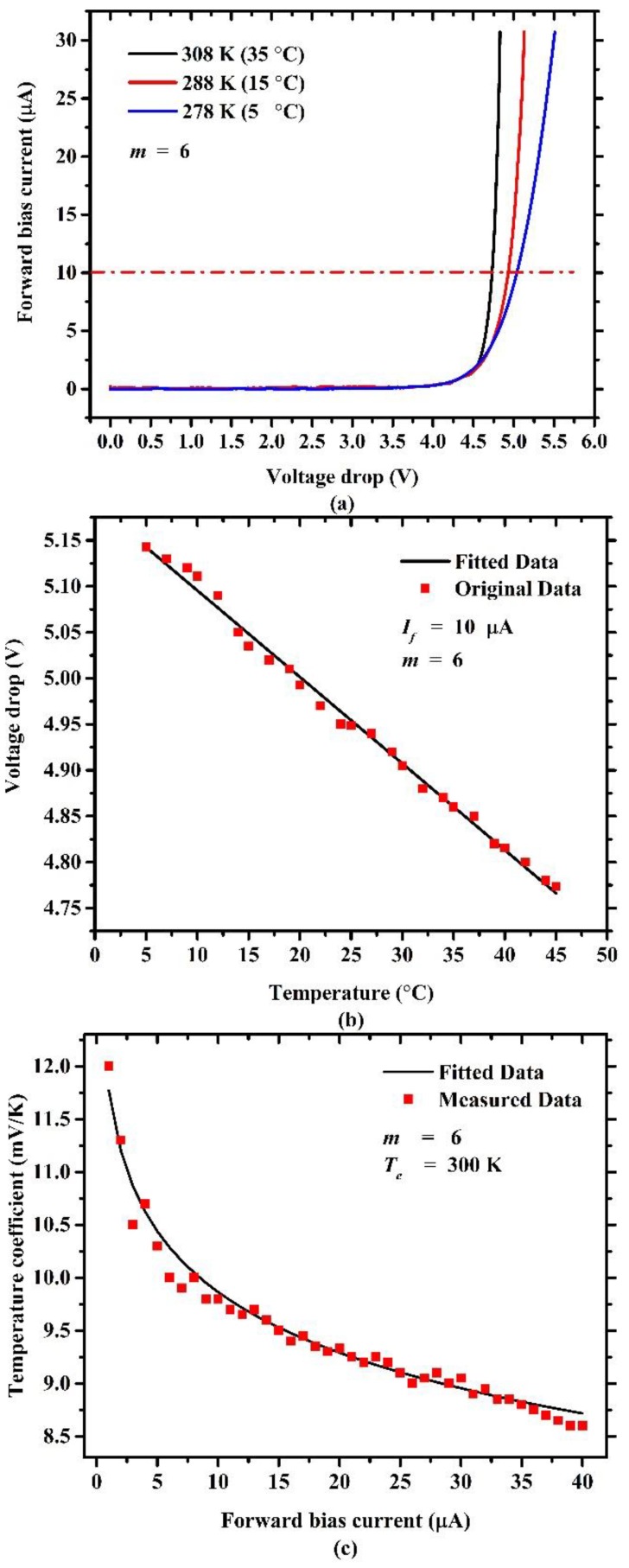
(**a**) Current–voltage (I–V) characteristics dependence on temperature. (**b**) Voltage drop dependence on temperature. (**c**) Temperature coefficient dependence on forward bias current of six series diodes.

This micro-Pirani vacuum sensor was based on the temperature coefficient of series diodes, and temperature coefficient had the greatest impact on voltage drop variation. To perform temperature coefficient measurements, a probe station system (SemiProbe SAVP-8, SemiProbe, Winooski, VT, USA) and a semiconductor analyzer (Agilent B1500A, Agilent Santa Rosa, CA, USA) were used. The sensor was placed on the chuck of the probe station system, and the temperature of the chuck was controlled by a temperature controller system. The temperature between the chuck and the sensor should be the same, thus that the temperature of the sensor can be controlled by the temperature controller. Once the sensor is released, temperature variation is largely due to the self-heating effect of the series diodes, which can result in temperature coefficient measurement errors. For accuracy, the measurement was carried out before the gap between the MHP and the substrate was released. First, the current–voltage (I–V) characteristics were measured to make sure that the series diodes worked. Next, the temperature controller of the probe station system controlled the temperature to the target temperature. Then, the voltage drop across series diodes was measured under the condition of a constant forward bias current by using the voltage–time (V–T) sampling mode of the Agilent B1500A. After that, the operation of changing the temperature and measuring the voltage drop was repeated. Finally, the measured data were processed to analyze the temperature coefficient of the series diodes. Figure 10a shows the measured I–V characteristics of the six series diodes with three different temperatures, and it is obvious that the I–V curves shifted left as the temperature rose. Figure 10b shows the measured voltage drops under the condition of 10-μA forward bias current with different temperatures. It can be seen that the voltage drops across the series diodes depended almost linearly on temperature. Figure 10c illustrates that the temperature coefficient was inversely proportional to the forward bias current. Under 10-μA forward bias current condition, the series diodes had a temperature coefficient of −9.5 mV/K.

**Figure 11:**
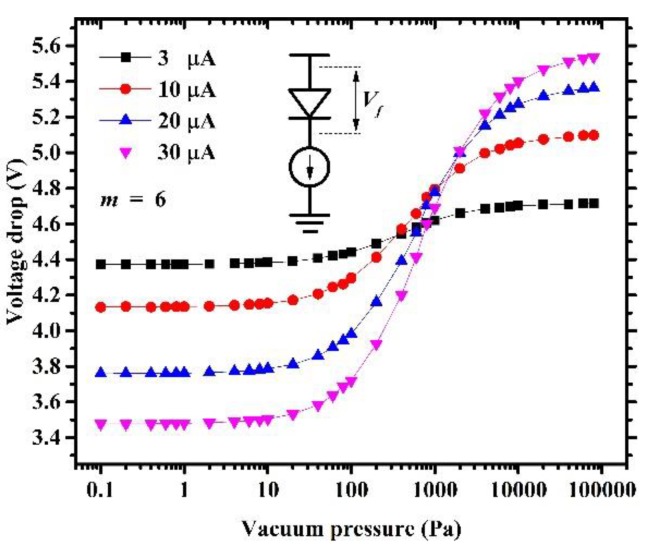
Voltage drop across six series diodes is a function of vacuum pressure and forward bias current.

The dynamic vacuum pressure range is the most important parameter of a micro-Pirani vacuum sensor. To inspect this parameter of the fabricated micro-Pirani vacuum sensor, the SemiProbe SAVP-8 probe station system and Agilent B1500A were used again. The sensor that was used for this measurement must be released. The vacuum pressure range of the probe station varied from 5 × 10^−3^ Pa to atmospheric pressure. A 10-μA forward bias current is a typical condition for this micro-Pirani vacuum sensor, and the performance of the sensor was analyzed under this condition. In addition, the measurements were also carried out under the condition of some other forward bias currents. Figure 11 shows the measured relationship between the voltage drop, forward bias current, and vacuum pressure. In a vacuum pressure range that was lower than 10^−1^ Pa, gaseous heat loss variation was not obvious and the voltage drop remained almost constant. In the vacuum pressure range between 10^−1^ and 10^4^ Pa, the voltage drop rose rapidly with the vacuum pressure because of the obvious increasing gaseous heat loss. When the vacuum pressure was higher than 10^4^ Pa, the gaseous heat loss was so high that the vacuum pressure had less influence on it, resulting in the voltage drop remaining nearly unchanged. Therefore, the dynamic vacuum pressure range of this series-diode-based micro-Pirani vacuum sensor was from 10^−1^ to 10^4^ Pa at the forward bias current of 10 μA. The average sensitivity was about 90 μV/Pa and its power consumption was only 50 μW.

It also can be seen that the performance of the sensor was improved by applying a greater forward bias current. When the series-diode-based micro-Pirani vacuum sensor worked at a 30-μA forward bias current, the dynamic vacuum pressure range expanded to the range of 10^−1^ to about 2 × 10^4^ Pa, and the average sensitivity reached 200 μV/Pa with a power consumption of 130 μW.

**Figure 12:**
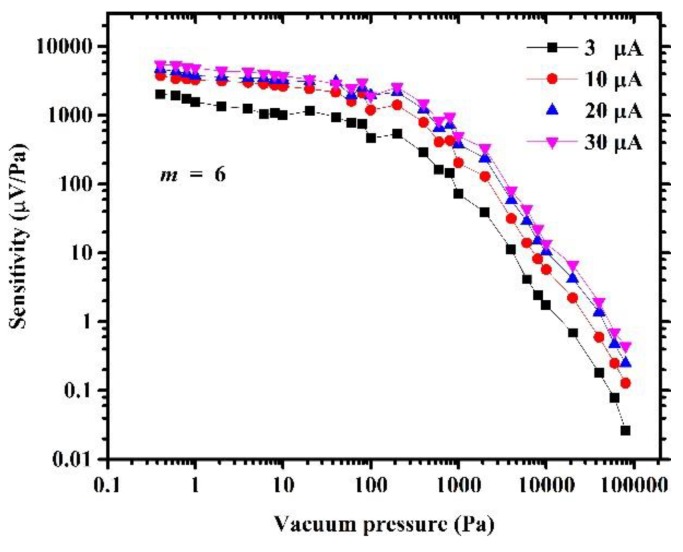
Sensitivity of the six-series-diode-based micro-Pirani vacuum sensor is proportional to the forward bias current and inversely proportional to vacuum pressure.

Figure 12 shows the sensitivity of the micro-Pirani vacuum sensor varied with vacuum pressure and forward bias current. The sensitivity was inversely proportional to the vacuum pressure. This can be explained by the fact that there is a decreasing influence of vacuum pressure on gaseous heat loss as the vacuum pressure increases, except that, sensitivity was proportional to the forward bias current. The greater the forward bias current, the higher the sensitivity. However, the forward bias current should be limited to avoid sensor failure because of the very high temperature of the MHP under the condition of low vacuum pressure.

A comparison of our work with some previous works is presented in Table 2. All these sensors are CMOS compatible. Some of the average sensitivity, sensor power consumption, the sensitivity of unit power consumption, and sensor size parameters are approximately calculated results from the data in the corresponding paper. It can be seen that the presented series-diode-based micro-Pirani vacuum sensor has the lowest power consumption (50 μW) at a forward bias current of 10 μA. Good dynamic vacuum pressure range and sensitivity are obtained based on the power consumption. Furthermore, the presented micro-Pirani vacuum sensor has the largest sensitivity of unit power consumption, which means that this series-diode-based micro-Pirani vacuum sensor is suitable for low power consumption designs.

## 5. Conclusions

This paper reports a highly sensitive, diode-based micro-Pirani vacuum sensor with low power consumption. The theoretical analysis and simulation were carried out, and optimization was made on two aspects based on the analysis and simulation. On the one hand, the temperature coefficient was enhanced by taking advantage of the temperature coefficient of the series diodes. On the other hand, temperature variation was enlarged by improving the sensor structure. These two factors mentioned above led to larger voltage drop variation and thus higher sensitivity. Finally, measurements of the series-diode-based micro-Pirani vacuum sensor were performed after optimization and fabrication. Test results showed that the series-diode-based micro-Pirani vacuum sensor had great performance. When the sensor was forward biased at a constant current of 10 μA with a power consumption as low as 50 μW, the dynamic vacuum pressure range was from 10^−1^ to 10^4^ Pa, average sensitivity was as high as 90 μV/Pa, and the sensitivity of unit power consumption increased to 1.8 V/W/Pa. In addition, the dynamic vacuum pressure range and average sensitivity could be promoted with an appropriately greater forward bias current. Therefore, the presented series-diode-based micro-Pirani vacuum sensor is a good candidate for vacuum pressure monitoring with low power consumption.

## Figures and Tables

**Table 1:** Redesigned geometries of the micro-Pirani vacuum sensor.

Total Size	Microhotplate Size (*L* × *W* × *H*)	Cantilever Size (*L_can_* × *N* × *M*)	Gap between MHP and Substrate
35 × 35 μm	16.5 × 16.5 × 2 μm	39 × 2 × 2 μm	22 μm

**Table 2:** Comparison of our work with some previous works.

Researcher	Average Sensitivity (μV/Pa) Sensitivity of Unit Power Consumption (V/W/Pa)	Dynamic Vacuum Pressure Range (Pa)	Sensor Power Consumption (μW)	Sensor Size (μm^2^)
J. Wang et al. [19]	230 μV/Pa (0.02 V/W/Pa)	1 to 1 × 10^2^	4900	100 × 100
Y. C. Sun et al. [17]	-	26.6 to 2.66 × 10^4^	127.59	206 × 82
M. Piotto et al. [10]	200 μV/Pa (0.18 V/W/Pa)	3 × 10^−1^ to 1 × 10^5^	1100	200 × 200
X. Sun et al. [29]	4.5 μV/Pa (0.001 V/W/Pa)	5 × 10^−3^ to 1 × 10^5^	4500	400 × 1500
F. Zhang et al. [30]	60 μV/Pa (0.43 V/W/Pa)	5 × 10^−3^ to 1 × 10^3^	140	500 × 500
Presented	90 μV/Pa (1.8 V/W/Pa)	1 × 10^−1^ to 1 × 10^4^	50	35 × 35
